# Efficacy of MAPK inhibitors in children with neurodegenerative Langerhans cell histiocytosis: Results from the European Consortium for Histiocytosis

**DOI:** 10.1002/hem3.70392

**Published:** 2026-06-07

**Authors:** Francesco Pegoraro, Francois Chalard, Dmitry Evseev, Mohamed Barkaoui, Solenne Le Louet, Julian Thalhammer, Nathalie Aladjidi, Alexandra Salmon, Perrine Marec‐Berard, Stefania Gaspari, Carmela De Fusco, Irene Trambusti, Antonio Verrico, Anna Raciborska, Karel Svojgr, Zdenka Křenová, Olga Slater, Gerard Millen, Myriam Ben Arush, Dana Ashkenazi Lustig, Tatiana Von Bahr Greenwood, Caroline Hutter, Itziar Astigarraga, Mónica López‐Duarte, Lorena Valero‐Arrese, María José Ortega Acosta, Cor van den Bos, Leonie Naeije, Daniel Martin‐Munoz, Anna Perrone, Marzia Mortilla, Olivier Felician, Ahmed Idbaih, Sébastien Héritier, Vasanta Nanduri, Milen Minkov, Jan‐Inge Henter, Elena Sieni, Jean Donadieu

**Affiliations:** ^1^ Department of Experimental and Clinical Medicine University of Florence Florence Italy; ^2^ Department of Hematology and Oncology Meyer Children's Hospital IRCCS Florence Italy; ^3^ French Reference Center for Langerhans Cell Histiocytosis Trousseau Hospital Paris France; ^4^ Radiology Department Trousseau Hospital Paris France; ^5^ Department of Hematopoietic Stem Cell Transplantation Dmitriy Rogachev National Center for Pediatric Hematology, Oncology and Immunology Moscow Russia; ^6^ Department of Pediatrics and Adolescent Medicine, Division of Pediatric Oncology Kepler University Hospital Linz Austria; ^7^ Department of Pediatric Hematology Lille University Hospital Lille France; ^8^ Department of Pediatric Hematology and Oncology Bordeaux University Hospital Bordeaux France; ^9^ Department of Pediatric Hematology and Oncology Strasbourg University Hospital Strasbourg France; ^10^ Department of Paediatric Oncology Léon‐Bérard Centre Lyon France; ^11^ Pediatric Hematology Oncology, Cellular and Gene Therapy Bambino Gesù Children's Hospital IRCCS Rome Italy; ^12^ Department of Pediatric Oncology AORN Santobono‐Pausilipon Naples Italy; ^13^ Neuro‐Oncology Unit IRCCS Istituto Giannina Gaslini Genoa Italy; ^14^ Department of Oncology and Surgical Oncology for Children and Youth Institute of Mother and Child Warsaw Poland; ^15^ Department of Pediatric Hematology and Oncology University Hospital Motol and 2nd Faculty of Medicine Prague Czech Republic; ^16^ Department of Pediatric Hematology and Oncology University Hospital Brno Brno Czech Republic; ^17^ Department of Paediatric Hemato‐Oncology Great Ormond Street Hospital London UK; ^18^ Department of Paediatric Oncology Birmingham Children's Hospital Birmingham UK; ^19^ Pediatric Oncology Rambam Medical Center Haifa Israel; ^20^ Childhood Cancer Research Unit, Department of Women's and Children's Health Karolinska Institute Stockholm Sweden; ^21^ Astrid Lindgrens Children's Hospital Karolinska University Hospital Stockholm Sweden; ^22^ St. Anna Children's Cancer Research Institute Vienna Austria; ^23^ Department of Pediatrics and Adolescent Medicine, St Anna Kinderhospital Medical University of Vienna Vienna Austria; ^24^ Hospital Universitario de Cruces, IIS Biobizkaia Universidad País Vasco UPV/EHU, Unidad de Hematología y Oncología Pediátrica Barakaldo Spain; ^25^ Pediatric Hematology Unit, Hematology Department Hospital de Valdecilla‐IDIVAL Santander Spain; ^26^ Pediatric Haematology and Oncology Division Vall d'Hebron University Hospital Barcelona Spain; ^27^ Pediatric Haematology Unit Virgen de las Nieves University Hospital Granada Spain; ^28^ Department of Hemato‐Oncology Princess Máxima Center for Pediatric Oncology Utrecht The Netherlands; ^29^ Department of Neuroradiology Karolinska University Hospital Stockholm Sweden; ^30^ Radiology Unit Meyer Children's Hospital IRCCS Firenze Italy; ^31^ Neuroradiology Meyer Children's Hospital IRCCS Firenze Italy; ^32^ Aix‐Marseille University, INSERM, INS Institut de Neurosciences des Systèmes Marseille France; ^33^ Neurology Unit Pitié Salpêtrière University Hospital Paris France; ^34^ Department of Paediatrics Watford General Hospital Watford UK

## Abstract

Neurodegenerative Langerhans cell histiocytosis (ND‐LCH) is a severe and debilitating complication of LCH with no effective treatment. In this study, we assessed the efficacy of MAPK inhibitors (MAPKi) in a large cohort of children with ND‐LCH. We included pediatric patients with ND‐LCH who received MAPKi between 2013 and 2025. Clinical and radiological responses were evaluated using validated and newly developed scoring systems. We included 60 patients and assessed the response to MAPKi in 54 (clinical *n* = 53, radiological *n* = 54). Of the 53 clinically evaluable patients, 44 (83%) presented with symptoms (impaired global functioning *n* = 39, 74%; neurological manifestations *n* = 38, 72%; and psychiatric disturbances *n* = 27, 51%). MAPKi were started at a median of 25 months (interquartile range [IQR] 5–67) from ND diagnosis. Clinical responses were observed in 29 patients (55%), while the remaining showed disease stabilization (*n* = 21, 39%) or progression (*n* = 3, 6%). Of the 54 patients with magnetic resonance imaging (MRI) follow‐up, radiological improvement was documented in 40 (74%), while 12 (22%) demonstrated stable findings, and 2 (4%) showed progression. We observed significant improvements in both clinical (P < 0.001) and MRI abnormalities (P < 0.001). None of the nine asymptomatic patients developed symptoms, while MRI lesions improved in six of them. Positive responses occurred irrespective of baseline disease severity and treatment delay. Adverse events, evaluable in 60 patients, were reported in 31 of them (52%) but were generally not severe and manageable. In conclusion, we demonstrated robust efficacy of MAPKi in ND‐LCH, with an acceptable safety profile. These findings support a promising therapeutic approach that warrants prospective validation in this challenging condition.

## INTRODUCTION

Neurodegenerative Langerhans cell histiocytosis (ND‐LCH) is one of the most challenging complications of LCH, occurring in up to 20% of children.^1^, ^2^, ^3^, ^4^, ^5^ Somatic mutations such as *BRAF* ^*V600E*^ are likely involved in ND pathogenesis, but the specific mechanisms are still debated.^6^, ^7^, ^8^ Clinically, ND‐LCH can be divided into two different entities: LCH‐associated abnormal central nervous system (CNS) imaging (LACI), consisting only of radiologic abnormalities that include magnetic resonance imaging (MRI) signal abnormalities mostly involving the cerebellum, the basal ganglia, and the pons, sometimes leading to atrophy; and LCH‐associated abnormal CNS symptoms (LACS), which develop in some patients with LACI and include neurologic (mostly cerebellar), cognitive, and psychological abnormalities, sometimes resulting in severe disability and death.^9^, ^10^

Despite recent therapeutic advances in LCH, ND remains a difficult‐to‐treat disease, with very limited and often ineffective treatment options. Historically, different approaches such as chemotherapy and immunomodulatory drugs have been attempted but have always failed to mitigate disease progression or improve symptoms.^11^, ^12^, ^13^, ^14^, ^15^ More recently, MAPK inhibitors (MAPKi) such as BRAF and MEK inhibitors (BRAFi, e.g., vemurafenib and dabrafenib; MEKi, e.g., cobimetinib and trametinib) have shown promising results in preclinical models of ND‐LCH and in very limited cohorts of pediatric and adult LCH,^16^, ^17^, ^18^, ^19^ and comprehensive data on their efficacy are lacking.

Here, we retrospectively analyzed a large cohort of patients with childhood‐onset ND‐LCH who were followed at centers of the European Consortium for Histiocytosis (ECHO) network and received MAPKi for the treatment of ND‐LCH. We analyzed responses to MAPKi, assessed using clinical and radiologic criteria developed and validated within the ECHO network.

## METHODS

### Patients

We retrospectively screened patients with biopsy‐proven LCH who developed radiologically confirmed childhood‐onset ND‐LCH. Patients who received MAPKi for the treatment of ND‐LCH between 2013 and 2025 were considered for study inclusion. We enrolled patients with neurodegeneration who received MAPKi and evaluated responses in those who completed at least six consecutive months of treatment and had available clinical and MRI data for response assessment. We enrolled both patients for whom the clinical ND was the main indication of MAPKi treatment (LACS) or patients with radiological ND without clinical symptoms for which MAPKi were indicated for refractory systemic LCH with LACI. Patients who developed ND‐LCH while receiving or just after discontinuing MAPKi (secondary ND‐LCH) were excluded from this study.

The initial screening was made among patients enrolled in a large retrospective ECHO study on the efficacy of MAPKi in pediatric LCH.^20^ Subsequently, a study proposal was circulated within the ECHO network, and national coordinators for histiocytosis were contacted for additional recruitment.

Patients were included in their respective national LCH registries, when available, and informed consent was obtained from their parents for enrollment in an observational study on MAPKi in pediatric LCH (PI Jean Donadieu). The study was conducted in accordance with the Declaration of Helsinki and its later amendments.^21^

### Data collection and outcomes

Data were collected from clinical charts by local physicians and were then stored in a password‐protected online repository held at the French Referral Center for Histiocytosis, as previously reported.^20^ The data collected related to demographics, clinical presentation of LCH, somatic mutations, ND‐related symptoms, treatment (type and dose of MAPKi, drugs used before or after MAPKi), toxicity, and responses, assessed using both validated and newly developed scales.

Outcomes included the following: (a) the assessment of the clinical response to MAPKi in patients treated for ND‐LCH in a comprehensive way that considers the whole spectrum of the disease (global functioning, neurologic symptoms, and psychiatric issues); (b) the assessment of the radiologic response to MAPKi in patients treated for ND‐LCH using a semiquantitative scale; (c) safety and tolerability of MAPKi in patients treated for ND‐LCH; and (d) potential predictors of response to MAPKi.

Adverse events (AEs) were evaluated by local clinicians based on the full review of medical charts. We included in the analyses all AEs related or possibly related to treatment, also including results of laboratory tests.

### Clinical response assessment

The clinical response was assessed using two different disability scales. First, we utilized the “Neurological” and “Education/employment/psychological” items from the disability score published by Nanduri et al.^22^ To encompass some of the limitations of this score (i.e., not comprehensive of the disease complexity, unable to detect minor but significant changes), we developed a specific scale (Figure S1) based on three different categories (global functioning, five items; neurologic symptoms, four items; and psychologic/psychiatric abnormalities, four items). Each of the items was scored from 0 to 2, in order to longitudinally estimate the progression or regression of symptoms in an individual patient.

The scale was designed to be easy to use for the retrospective collection of data (5–10 min), comprehensive of all aspects of ND‐LCH, and capable of identifying significant clinical changes (any score change implies significant changes for the patient). The scale was conceived by F.P. and J.D. and then circulated within the ECHO network for refinement and content‐validation. The scale was named after the grant from the Histiocytose France Association (dedicated to a patient named Florent) that supported this project. Hereafter referred to as the “Florent scale,” it was administered to patients and caregivers by local clinicians at the last follow‐up and retrospectively at MAPKi start, and further verified through chart review when available (i.e., as a combination of medical records and delayed recall of proxies). Patients with a global score of 0 were considered asymptomatic, patients with a score from 1 to 3 were classified as having mild ND, whereas those with a Florent score ≥ 4 were classified as having moderate/severe ND. Responses were assessed using both the disability score by Nanduri et al.^22^ and the Florent scale, and we considered as a response each decrease of these scores in any domain.

### Radiological response assessment

The neuroradiological response was assessed using a modified version of the scale proposed by Prosch et al.^23^ The modified scale included an MRI assessment of signal abnormalities and atrophy in five anatomical brain regions, applying a semiquantitative evaluation. Specifically, signal intensity was scored from 0 to 3 in each anatomical region (Figure S2). Mandatory time‐points for MRI image evaluation were (a) MAPKi initiation and (b) last follow‐up on MAPKi. MRIs performed at the last follow‐up in patients who discontinued MAPKi (c) were also collected in patients with a follow‐up duration > 1 year after MAPKi discontinuation. The scale used for the study was validated within the radiology working group of the ECHO CNS group. All images were centrally reviewed in Paris by FC, the French referral neuroradiologist for LCH with more than 10‐year experience.

Regarding response definition, any decrease in signal intensity on the MRI scale between baseline and follow‐up imaging was considered indicative of a radiologic response. Overall radiologic response was also assessed while accounting for the presence of atrophy, to ensure robustness of the results, as atrophy may develop in patients whose signal abnormalities improve with treatment.

### Statistical analysis

Categorical variables are reported as numbers and percentages, whereas continuous variables are reported as medians and interquartile ranges (IQRs). Comparisons between groups were made using the Mann–Whitney *U* test for continuous variables and the chi‐square test for categorical variables. Variations in the clinical and radiological scales from treatment initiation to last follow‐up were compared using the Wilcoxon signed‐rank test. Clinical and radiological progression‐free survival probabilities were estimated using the Kaplan–Meier method. A composite response measure was defined as having a clinical response or remaining stable with radiological response. A two‐sided P‐value ≤ 0.05 was considered to indicate statistical significance. Statistical analyses were performed using STATA for Windows (version 18).

## RESULTS

**Figure 1:**
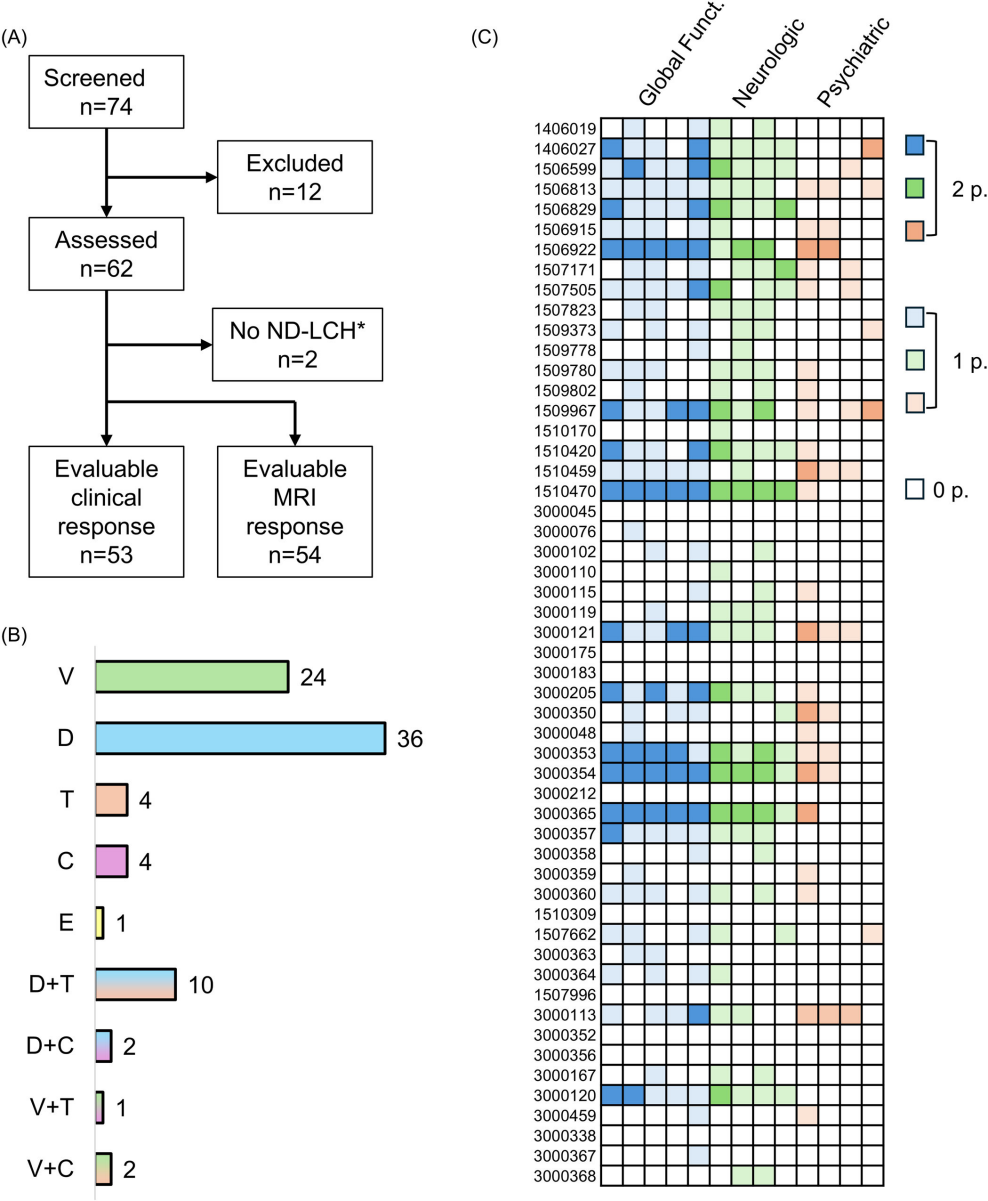
**Patient inclusion, clinical presentation, and treatment. (A)** Flow chart of patient inclusion; **(B)** therapeutic instances in the 60 assessed patients with neurodegenerative Langerhans cell histiocytosis (ND‐LCH); and **(C)** clinical presentation of ND‐LCH in the 53 patients assessed for clinical response. Each column represents an item of the score: global functioning in blue (autonomy, weakness, daily activities, social abilities, and school/work), neurologic symptoms in green (walking, speech, fine movements, and swallowing), and psychologic/psychiatric abnormalities in orange (emotional dysregulation, psychiatric symptoms, aggressiveness, and addiction). *After the central revision of magnetic resonance imaging (MRI) images. C, cobimetinib; D, dabrafenib; E, encorafenib; T, trametinib; V, vemurafenib.

Seventy‐four patients from 13 countries were screened for study inclusion (Figure 1A, Table S1). Eight were excluded for insufficient available data, two for lack of follow‐up, one for lack of histology, and one because the parents chose to interrupt treatment one week after initiation. Finally, 62 patients were assessed (39 males and 23 females), but in 2 of them, ND‐LCH was excluded at centralized revision (mild MRI abnormalities were not considered consistent with ND‐LCH). Of the 60 included patients, 54 (90%) received MAPKi for at least 6 months, and were evaluable for response assessment (53 clinical and 54 radiologic responses available).

### Clinical characteristics and treatment

The clinical characteristics of the 54 patients with response assessment are detailed in Table 1. The median age at LCH diagnosis was 25 months (IQR 11–40). At LCH diagnosis, 6 patients (11%) had single‐system disease, 35 (65%) multisystem risk‐organ negative involvement, and 13 (24%) risk‐organ involvement. Craniofacial bone lesions were detected in 39 patients (72%) and diabetes insipidus in 23 (43%). Almost all tested patients (*n* = 51/52, 98%) harbored the *BRAF* ^*V600E*^ mutation, and no mutation other than *BRAF* ^*V600E*^ was identified. The median age at ND diagnosis was 71 months (IQR 41–101), with a median delay after LCH diagnosis of 46 months (11–76).

**Table 1:** Clinical presentation of the 54 patients with response assessment either clinically or radiologically.

	Total *n* = 54
Age at LCH diagnosis, months—median (IQR)	25 (11–40)
Female sex—*n* (%)	18 (33)
Disease classification at diagnosis—*n* (%)	
SS	6 (11)
MS RO−	35 (65)
MS RO+	13 (24)
Organ involvement—*n* (%)	
Bone	46 (85)
Craniofacial bone lesions	39 (72)
Skin/mucosal	27 (50)
BM	5 (9)
Liver	10 (19)
Spleen	5 (9)
Lung	5 (9)
LN	6 (11)
Pituitary	23 (43)
CNS mass	6 (11)
*BRAF* ^*V600E*^ mutation	51/52 (98)
Age at ND diagnosis, months—median (IQR)	71 (41–101)
Time from LCH diagnosis to ND, months—median (IQR)	46 (11–76)
ND clinical presentation (*n* = 53)	
Asymptomatic	9 (17)
Global functioning impairment	39 (74)
Neurologic symptoms	38 (72)
Psychiatric symptoms	27 (51)
Time from ND to MAPKi initiation, months—median (IQR)	25 (5–67)
Age at MAPKi initiation, months—median (IQR)	113 (66–180)
Follow‐up since MAPKi initiation, months—median (IQR)	36 (16–64)

Abbreviations: BM, bone marrow; CNS, central nervous system; IQR, interquartile range; LCH, Langerhans cell histiocytosis; LN, lymph nodes; MAPKi, MAPK inhibitors; MS, multisystem; ND, neurodegeneration; RO, risk‐organ; SS, single‐system.

**Figure 2:**
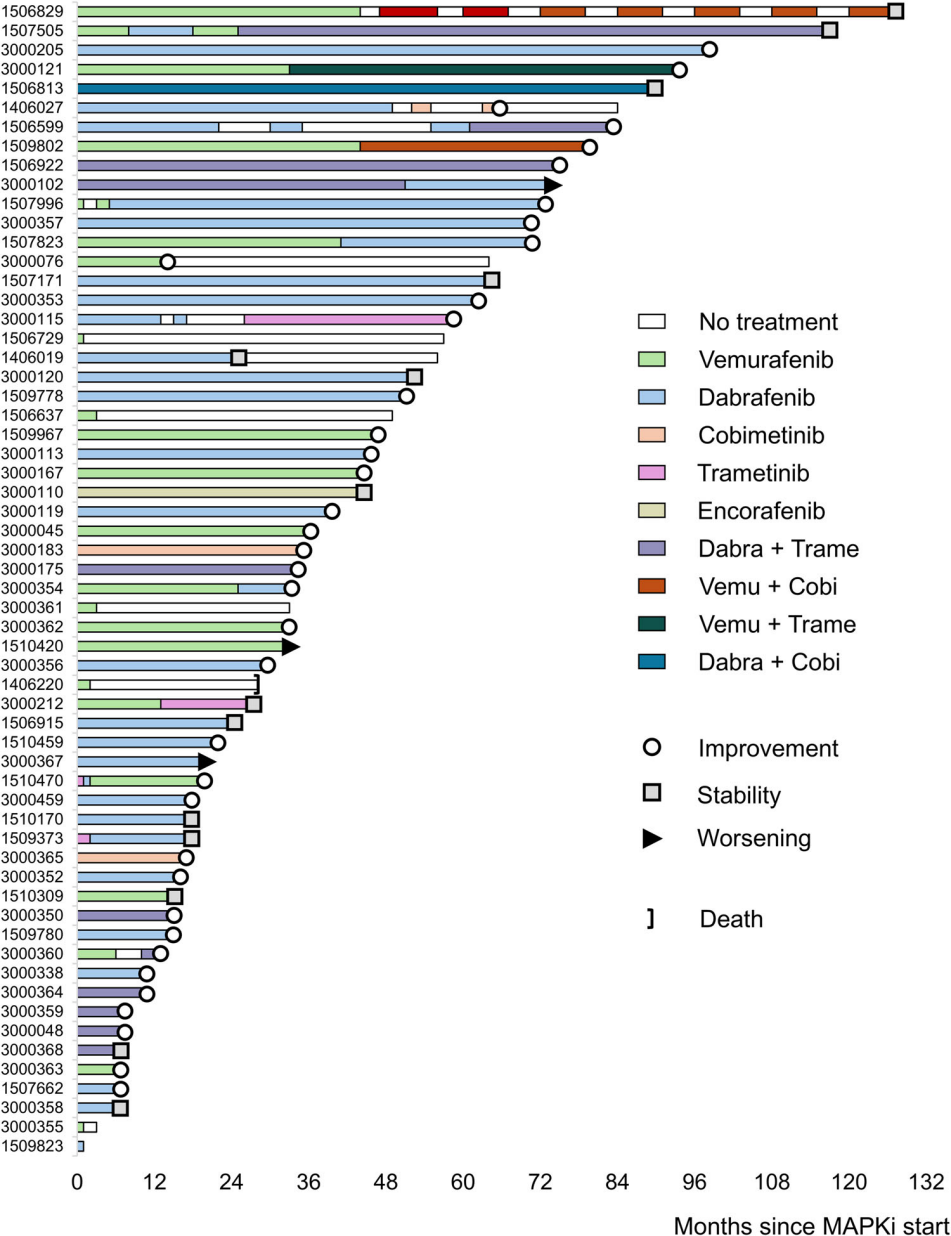
**Swimmer plot of treatment and response in the 60 patients with neurodegenerative Langerhans cell histiocytosis (ND‐LCH) who received MAPK inhibitors (MAPKi).** We considered responders those patients who had clinical improvement or remained asymptomatic with magnetic resonance imaging (MRI) improvement at the last follow‐up (except for NUP3000362, who had no clinical assessment and only a radiologic response). In six patients, response was not assessed due to treatment duration < 6 months.

A total of 84 therapeutic instances were reported in the 60 assessed patients with ND‐LCH (Figure 1B), with a median delay from ND diagnosis of 25 months (IQR 5–67) and a median age at MAPKi start of 113 months (IQR 66–180). They included: dabrafenib (*n* = 36, 43%); vemurafenib (*n* = 24, 29%); dabrafenib + trametinib (*n* = 10, 12%); trametinib and cobimetinib (*n* = 4 each, 5%); dabrafenib + cobimetinib and vemurafenib + cobimetinib (*n* = 2 each, 2%); and encorafenib or vemurafenib + trametinib (*n* = 1 each, 1%). Forty‐five patients (75%) received only one line of treatment, while the remaining 15 patients received two (*n* = 10) or more (*n* = 5) lines, due to toxicity or suboptimal response (Figure 2). Treatments were chosen by local physicians according to availability and local practice. Of note, seventeen patients (28%) received one or more lines of treatment for ND‐LCH prior to MAPKi, including high‐dose chemotherapy in eight, retinoic acid in six, and intravenous immunoglobulins in six.

Of the 53 patients with available clinical scores, nine (17%) were asymptomatic at MAPKi start (i.e., LACI, Florent score = 0). The indication of MAPKi in this group of patients was systemic refractory LCH. Of the remaining 44 symptomatic patients, 39 (74%) had impairment of global functioning, 38 (72%) had neurologic symptoms, and 27 (51%) had psychiatric symptoms (Table 1). The ND presentation at treatment start in these 53 patients is reported in Figure 1C.

### Response to treatment

**Figure 3:**
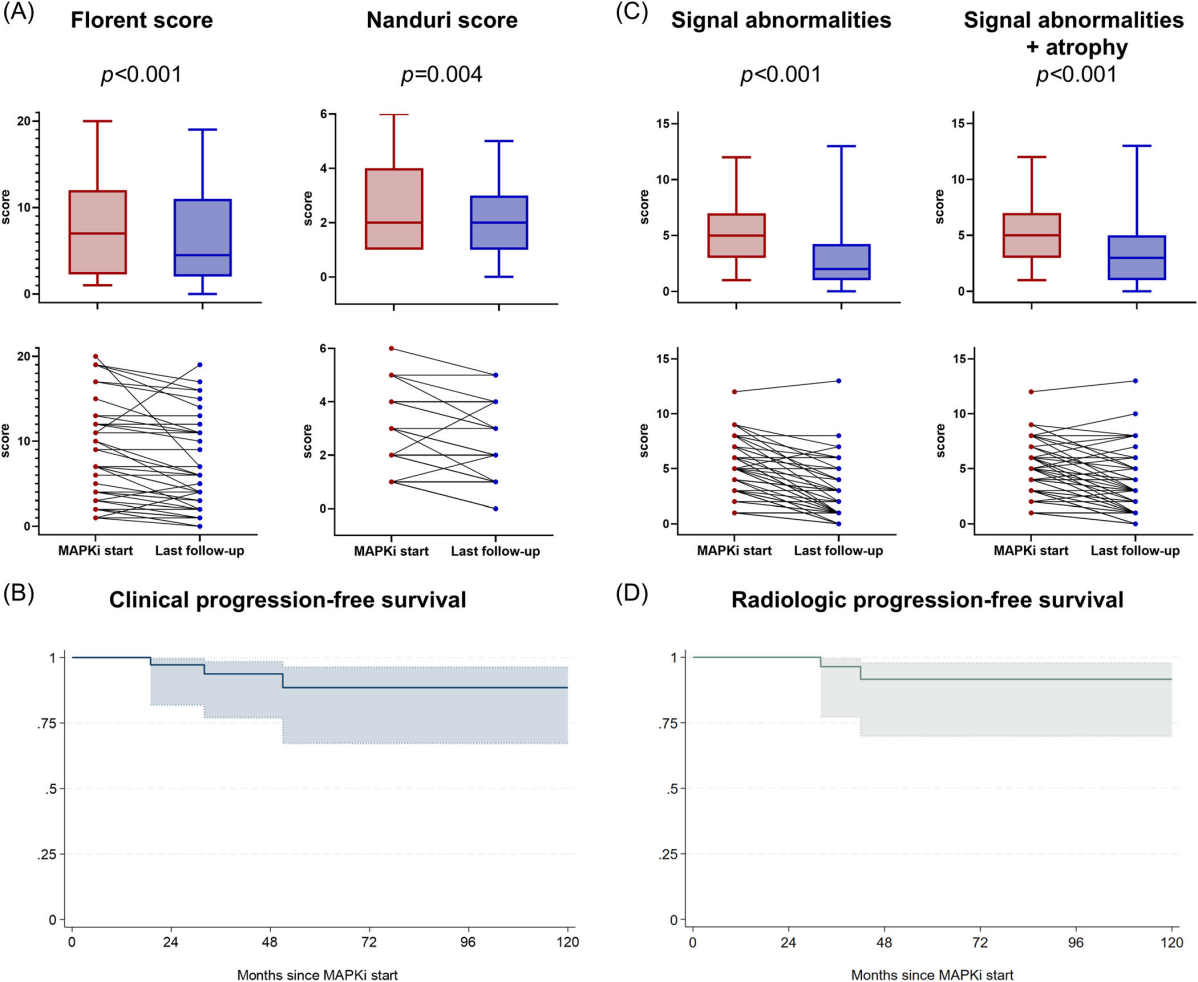
**Clinical and radiologic responses to MAPK inhibitors (MAPKi) in the 54 evaluable patients. (A)** Variations in the Florent and Nanduri score between MAPKi start and last follow‐up on treatment in the 44 symptomatic patients; **(B)** estimated clinical progression‐free survival probability; **(C)** variations in the radiologic score (without and with atrophy) between MAPKi start and last follow‐up on treatment in the 54 assessed patients; and **(D)** estimated radiologic progression‐free survival probability.

After a median treatment duration of 36 months (IQR 16–64), we were able to assess the clinical response in 53 patients (85%) and the radiological response in 54 (87%). Among the 53 clinically assessable patients, 29 patients (55%) had a clinical improvement, while 21 (39%) remained stable, and 3 (6%) progressed. Of the nine patients asymptomatic at treatment start, none developed symptoms during treatment, while MRI lesions improved in six of them. In symptomatic patients, the clinical response rate was 66% (*n* = 29/44), and we observed a statistically significant reduction of 2.5 points in the median Florent score (P < 0.001; Figure 3A). Similarly, using the Nanduri score, 18 (34%) patients improved, 32 (60%) remained stable, and 3 (6%) worsened, with a statistically significant difference between pre‐ and post‐treatment (P = 0.004; Figure 3A). The overall 5‐year estimated clinical progression‐free survival probability was 88% (95% CI 66–96; Figure 3B).

**Figure 4:**
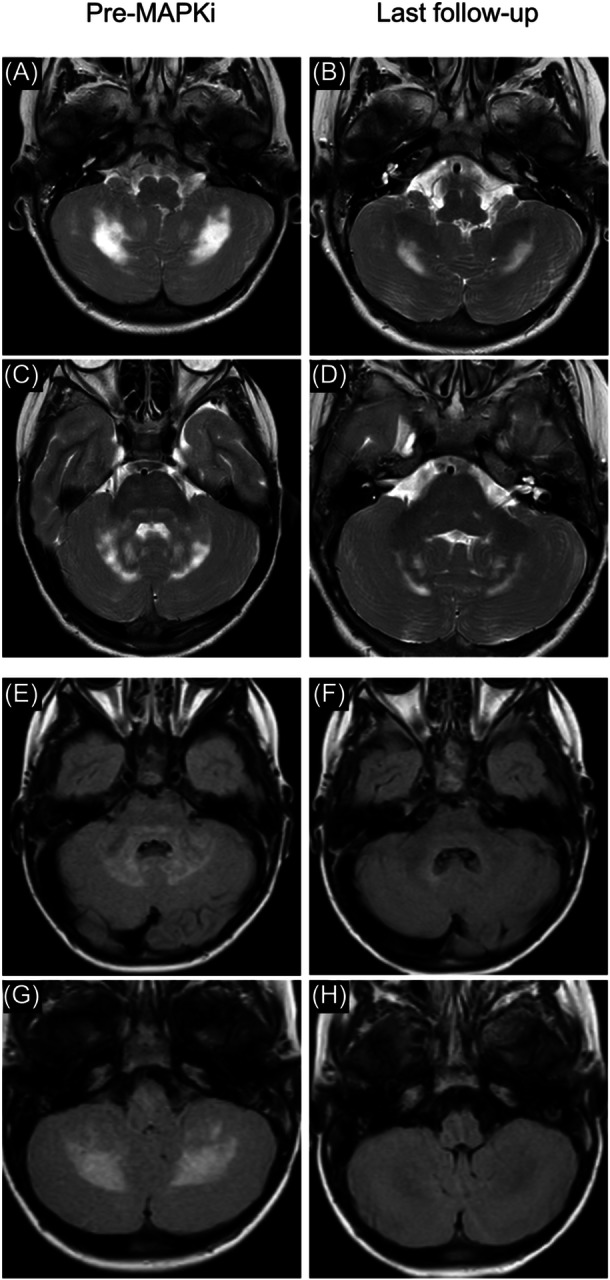
**Magnetic resonance imaging response to MAPK inhibitors (MAPKi) of two different patients before treatment initiation, compared with the last follow‐up on treatment. (A–D)** Axial T2 images showing areas of hypersignal involving the cerebellar white matter **(A)**, with partial regression of the lesions after MAPKi treatment **(B)**; and areas of hypersignal involving the dentate nuclei, cerebellar white matter, and dorsal pons **(C)**, with complete regression of the lesions in the dorsal pons and partial regression in the dentate nuclei and cerebellar white matter after MAPKi treatment **(D)**. **(E–H)** Axial FLAIR images showing areas of hypersignal involving the dentate nuclei, the cerebellar white matter, and the dorsal pons **(E)**, with almost complete regression of the lesions after MAPKi treatment **(F)**; and areas of hypersignal involving the cerebellar white matter **(G)** with complete regression of the lesions after MAPKi treatment **(H)**.

Among the 54 radiologically assessable patients, MRI signal abnormality improvement was observed in 40 patients (74%), while 12 (22%) remained stable, and 2 (4%) showed a mild worsening, with a statistically significant median reduction of 3 points (P < 0.001; Figure 3C). The same was also confirmed after considering atrophic changes in the scale (median reduction 2 points, P < 0.001; Figure 3C). The 5‐year estimated radiologic progression‐free survival probability was 91% (95% CI 70–98; Figure 3D). Examples of MRI response are depicted in Figure 4.

### Survival

Survival was assessed in the 60 patients who received MAPKi for ND‐LCH. After a median follow‐up of 36 months (IQR 16–64) since MAPKi initiation, 61 patients (98%) were alive. Patient NUP1406220 received vemurafenib for only 2 months before discontinuation due to toxicity; following cessation of vemurafenib, the patient developed progressive worsening of cerebellar symptoms, ultimately leading to death 26 months after BRAFi withdrawal. The 5‐year estimated probability of survival after MAPKi was 97% (95% CI 82–99; Figure S3).

### Factors associated with outcome

In order to evaluate treatment efficacy regardless of the clinical presentation and ND disease severity, we defined responding patients as those who either had a clinical improvement or remained asymptomatic with MRI improvement at the last follow‐up. Overall, a composite response was observed in 37 patients (69%, Figure 2), including 29 clinical responders, nine asymptomatic patients with MRI responses, and one patient with no available Florent score but with a good MRI response. We also investigated whether the patients' baseline characteristics at ND diagnosis were associated with a global response to MAPKi, but no significant association was identified (Table S2).

We then evaluated if the clinical characteristics of the patients with a favorable outcome at last follow‐up (mild ND‐LCH, Florent score < 4) differed from those with a more severe burden of neurodegeneration (moderate‐severe ND‐LCH, Florent score ≥ 4). Notably, patients with a Florent score < 4 at the last follow‐up started MAPKi treatment with a significantly shorter interval from ND diagnosis compared to those with a score ≥ 4 (median time 12 vs. 49 months, P < 0.001). They also presented with a lower disease burden at treatment start, both clinically (median Florent score 1 vs. 11, P < 0.001) and radiologically (median MRI score 4 vs. 6, P = 0.039), consistent with less advanced ND‐LCH prior to treatment (Table 2).

**Table 2:** Differences between patients with mild neurodegenerative Langerhans cell histiocytosis (ND‐LCH) and moderate‐severe ND‐LCH at last follow‐up (in the 53 patients with available Florent score).

	Mild ND (Florent 0–3) (*n* = 26)	Moderate‐severe ND (Florent ≥ 4) (*n* = 27)	P‐value
Age at ND diagnosis, months—median (IQR)	64 (27–95)	95 (57–122)	0.092
Time from LCH diagnosis to ND, months—median (IQR)	36 (22–72)	54 (11–82)	0.528
Time from ND to MAPKi initiation, months—median (IQR)	12 (2–34)	49 (16–98)	<0.001
Florent score pre‐treatment—median (IQR)	1 (0–3)	11 (7–15)	<0.001
MRI score pre‐treatment—median (IQR)	4 (2–6)	6 (4–8)	0.039
Response to treatment—*n* (%)	21 (81)	16 (59)	0.634
Treatment duration, months—median (IQR)	24 (12–42)	41 (19–75)	0.032

Abbreviations: IQR, interquartile range; LCH, Langerhans cell histiocytosis; MAPKi, MAPK inhibitors; ND, neurodegeneration.

### Tolerability

Tolerability was assessed in the 60 patients who received MAPKi for ND‐LCH, and AEs occurred in 31 of them (52%; Table 3). The most frequently reported included skin toxicities (e.g., photosensitivity, maculopapular or acneiform lesions) in 23 patients (38%), systemic (e.g., arthralgia/myalgia, fatigue) in 16 (27%), and gastrointestinal (e.g., abdominal pain, cholestasis) in five (8%). AEs were Grade 3–4 in 10 patients (17%), but in all cases, they resolved upon discontinuation. Nine patients (15%) required dose reduction due to toxicity. Drug withdrawal, due to clinician or family decision, occurred in 18 cases (30%): 12 of them permanently discontinued MAPKi (including 6 patients with treatment duration < 6 months for whom response assessment was not possible), while the remaining 6 were able to tolerate another MAPKi (Figure 2).

**Table 3:** Adverse events in the 60 included patients with confirmed neurodegenerative Langerhans cell histiocytosis (ND‐LCH).

	Total	Grade 1–2	Grade 3–4
At least one adverse event	31 (52)	21 (35)	10 (17)
Adverse events requiring treatment withdrawal	18 (30)	8 (13)	10 (17)
Adverse events requiring dose reduction	9 (15)	9 (15)	0 (0)
Skin			
Photosensitivity	9 (15)	6 (10)	3 (5)
Maculopapular lesions	7 (12)	7 (12)	0 (0)
Acne	3 (5)	2 (3)	1 (2)
Panniculitis	1 (2)	1 (2)	0 (0)
Angioedema	1 (2)	1 (2)	0 (0)
Nevus	1 (2)	1 (2)	0 (0)
Alopecia	1 (2)	1 (2)	0 (0)
Systemic			
Arthralgia/myalgia	8 (13)	8 (13)	0 (0)
Fatigue	5 (8)	4 (7)	1 (2)
Headache	3 (5)	3 (5)	0 (0)
Gastrointestinal			
Abdominal pain	2 (3)	2 (3)	0 (0)
Cholestasis	2 (3)	1 (2)	1 (2)
Diarrhea	1 (2)	1 (2)	0 (0)
Infections			
Mastoiditis	1 (2)	0 (0)	1 (2)
Paronychia	1 (2)	1 (2)	0 (0)
Cardiac			
Myocarditis	1 (2)	1 (2)	0 (0)
Ocular			
Exophthalmos	2 (3)	0 (0)	2 (3)
Hematologic			
Anemia	1 (2)	1 (2)	0 (0)
Endocrine			
SIADH	1 (2)	0 (0)	1 (2)

*Note*: Data are *n* (%).

Abbreviation: SIADH, syndrome of inappropriate antidiuretic hormone secretion.

## DISCUSSION

In this observational cohort study, we demonstrated the clinical and radiological response to MAPKi in 54 children with neurodegenerative LCH. Responses were observed regardless of age, duration of disease, type of inhibitor, and clinical severity, and treatments were usually well tolerated.

Neurodegeneration is a potentially devastating consequence of LCH, for which there is no proven effective treatment, and conventional therapy achieves, at best, only disease stabilization. The disease course is very heterogeneous, ranging from asymptomatic cases to a progressive disease with severe disability.^1^, ^2^, ^3^, ^4^, ^5^ Clinical trials and even observational studies evaluating therapies for ND‐LCH have been limited by very small sample sizes, short follow‐up durations, and heterogeneous assessment methods. Early studies included fewer than 10 patients, such as those investigating retinoic acid,^11^ vincristine plus cytarabine,^15^ and IVIG.^12^ The largest series assessing IVIG included 23 patients and ultimately concluded that this treatment lacked efficacy in preventing neurological progression.^5^ Finally, evidence for MAPKi therapy remains limited to anecdotal reports, typically involving fewer than five patients.^16^ For such reasons, this is the first description of the evaluation of the MAPKi effect in a large cohort of children with ND‐LCH.

As this series appears to be original, we were compelled to develop a dedicated set of evaluation criteria, including both a comprehensive clinical scoring system and a standardized MRI assessment. At present, external validation of these tools is not available. Nevertheless, longitudinal follow‐up was conducted using consistent methodologies, with all patients assessed using the same clinical scale and MRI protocol across sequential evaluations. Moreover, while the subject of introducing recall bias, the items included in the score were also reported in clinical charts. Overall, these tools might represent reliable and practical instruments that could enhance patient management in both real‐world practice and future clinical studies.

Globally, responses were observed both in patients with long‐lasting ND and severe clinical impairment, as well as in patients with mild symptoms. Even in patients with a very severe disease, the introduction of MAPKi resulted in at least disease stabilization, which is clinically relevant considering the progressive course of this condition. Of note, the only patient from this series who deceased had stopped MAPKi after a few months due to poor tolerance and chose not to receive any other MAPKi thereafter.

Comparison between different inhibitors was limited by the heterogeneity of the therapeutic instances, but no specific inhibitor appeared to be more effective, suggesting that treatment can be adapted to the expertise and availability of MAPKi at each center. All treatments were generally well tolerated, although discontinuations were more frequent than reported in multisystem LCH.^17^, ^24^ A discontinuation of one molecule was observed in 30% of patients, with 10% resuming a MAPKi and 20% definitively stopping treatment. However, some of these discontinuations occurred in the context of Grade 1–2 AEs and limited data regarding the efficacy of MAPKi in this setting at the time of treatment, which led some parents to request treatment discontinuation. Side effects were not uncommon either (52%), but the most severe ones resolved upon reduction of dose or discontinuation, and patients were usually able to mitigate toxicity through a switch to another inhibitor. In most cases, a certain degree of toxicity was generally accepted by clinicians and families, considering treatment efficacy and the lack of valuable alternatives. In this regard, possible alternative strategies, such as CSF1R inhibitors^8^ or intra‐arterial chemotherapy,^25^ have shown preclinical efficacy or encouraging efficacy in adults with histiocytosis, representing possible future approaches for pediatric ND‐LCH.

In this work, we could not demonstrate an association between shorter treatment delay and response to MAPKi. Clinical responses were observed both in patients with long‐standing ND and severe disease burden and in those with milder manifestations. However, outcomes at last follow‐up were more favorable in patients who initiated treatment earlier, likely because disease progression was less advanced at the time of treatment initiation. Although not among the objectives of our study, this finding reinforces the need for early recognition and treatment of this condition.

However, the optimal timing for treatment initiation remains uncertain. While therapy is clearly indicated in symptomatic patients due to demonstrated clinical efficacy, its use in asymptomatic patients with LACI remains controversial.^10^ Data from the Italian registry suggest that MRI progression correlates with clinical deterioration, indicating that treatment decisions in asymptomatic patients might be guided by evidence of MRI worsening.^5^ An alternative approach could be to initiate treatment early, with the aim of preventing MRI worsening since such worsening is likely associated with brain damage. Novel biomarkers such as cerebrospinal and plasma neurofilament light might additionally help tailor therapeutic decisions in asymptomatic patients.^18^, ^26^ Moreover, their reliability in response monitoring might represent another potential tool for guiding treatment duration, especially in the case of poor tolerability. Uncertainty regarding optimal treatment duration and the challenges associated with treatment discontinuation represent additional unresolved issues.

Patients with secondary ND‐LCH (i.e., those who developed ND‐LCH during or shortly after MAPKi therapy) were excluded because of the risk of significant confounding. This subgroup, only recently characterized,^20^, ^27^ differs clinically from patients with ND‐LCH at presentation, notably in terms of younger age at diagnosis and minimal pituitary involvement, and largely consists of individuals treated with MAPKi for refractory or risk‐organ–positive disease. Moreover, the occurrence of ND in this context may reflect emerging resistance to MAPKi. In contrast, all patients included in our study were MAPKi‐naïve at treatment initiation and displayed a classical ND‐LCH phenotype, supporting the methodological choice to exclude secondary cases.

Our study has limitations, mainly due to its retrospective design and the intrinsic heterogeneity of the collected data and follow‐up. In addition, the absence of pharmacokinetic data limited the assessment of exposure‐response relationships and interindividual variability in treatment outcomes. Of note, pediatric formulations of MAPKi are limited, and patients were therefore often required to rely on locally compounded preparations. Nonetheless, this work reflects a real‐world setting in which the efficacy of MAPKi was demonstrated under the best available clinical practice. Based on these findings, together with accumulating evidence on MAPKi efficacy in multisystem LCH,^20^ this consortium advocates that these agents become increasingly accessible to patients.

In conclusion, MAPKi proved to be effective and safe in children with ND‐LCH, irrespective of disease severity or therapeutic delay. Although validation in prospective clinical trials is still warranted, these findings offer encouraging prospects for patients and their caregivers.

## AUTHOR CONTRIBUTIONS

**Francesco Pegoraro**: Conceptualization; investigation; writing—original draft; methodology; validation; visualization; formal analysis; data curation; writing—review and editing. **Francois Chalard**: Investigation; visualization; writing—review and editing; formal analysis; data curation. **Dmitry Evseev**: Investigation; writing—review and editing. **Mohamed Barkaoui**: Investigation; writing—review and editing; project administration; data curation. **Solenne Le Louet**: Investigation; writing—review and editing. **Julian Thalhammer**: Investigation; writing—review and editing. **Nathalie Aladjidi**: Investigation; writing—review and editing. **Alexandra Salmon**: Investigation; writing—review and editing. **Perrine Marec‐Berard**: Investigation; writing—review and editing. **Stefania Gaspari**: Investigation; writing—review and editing. **Carmela De Fusco**: Investigation; writing—review and editing. **Irene Trambusti**: Investigation; writing—review and editing. **Antonio Verrico**: Investigation; writing—review and editing. **Anna Raciborska**: Investigation; writing—review and editing. **Karel Svojgr**: Investigation; writing—review and editing. **Zdenka Křenová**: Investigation; writing—review and editing. **Olga Slater**: Investigation; writing—review and editing. **Gerard Millen**: Investigation; writing—review and editing. **Myriam Ben Arush**: Investigation; writing—review and editing. **Dana Ashkenazi Lustig**: Investigation; writing—review and editing. **Tatiana Von Bahr Greenwood**: Investigation; writing—review and editing. **Caroline Hutter**: Investigation; writing—review and editing. **Itziar Astigarraga**: Investigation; writing—review and editing. **Mónica López‐Duarte**: Investigation; writing—review and editing. **Lorena Valero‐Arrese**: Investigation; writing—review and editing. **María José Ortega Acosta**: Investigation; writing—review and editing. **Cor van den Bos**: Investigation; writing—review and editing. **Leonie Naeije**: Investigation; writing—review and editing. **Daniel Martin‐Munoz**: Investigation; writing—review and editing. **Anna Perrone**: Investigation; writing—review and editing. **Marzia Mortilla**: Investigation; writing—review and editing. **Olivier Felician**: Investigation; writing—review and editing. **Ahmed Idbaih**: Investigation; writing—review and editing. **Sébastien Héritier**: Investigation; writing—review and editing. **Vasanta Nanduri**: Investigation; writing—review and editing. **Milen Minkov**: Investigation; writing—review and editing; resources. **Jan‐Inge Henter**: Conceptualization; investigation; writing—review and editing; validation; supervision; methodology. **Elena Sieni**: Conceptualization; investigation; writing—review and editing; validation; methodology; supervision. **Jean Donadieu**: Methodology; conceptualization; investigation; funding acquisition; writing—review and editing; validation; project administration; resources; supervision; data curation.

## CONFLICT OF INTEREST STATEMENT

The authors declare no conflicts of interest.

## ETHICS STATEMENT

IRB Île‐de‐France III (#2011‐A00447‐34; 2011‐2020) and IRB Sud‐Ouest et Outre‐Mer II (#2019‐A01814‐53; since 2020) (CNIL #909207).

## Supporting information

Supporting Information.

## Data Availability

The data that support the findings of this study are available on request from the corresponding author. The data are not publicly available due to privacy or ethical restrictions.
