# Supplementary material for: Efficacy of MAPK inhibitors in children with neurodegenerative Langerhans cell histiocytosis: Results from the European Consortium for Histiocytosis
Source: Hemasphere. 2026 Jun 7;10(6):e70392. doi: 10.1002/hem3.70392 (PMC13242968; doi:10.1002/hem3.70392)

**Table S1.** Included centers and patients’ inclusion

| **Country (Center)** | **Screened** | **Included** |
| --- | --- | --- |
| France  Trousseau Hospital (Paris)  Lyon University Hospital  Lille University Hospital  Bordeaux University Hospital  Rennes University Hospital  Grenoble University Hospital  Strasbourg University Hospital  Marseille University Hospital | 23  10  3  2  2  2  2  1  1 | **22**  10  2  2  2  2  2  1  1 |
| Italy  Meyer Children’s Hospital IRCCS (Florence)  Bambino Gesù Children’s Hospital IRCCS (Rome)  Santobono Children’s Hospital (Naples)  Gaslini Children’s Hospital IRCCS (Genoa)  Civico Di Cristina Benfratelli Hospital (Palermo) | 16  7  5  1  1  2 | **12**  6  4  1  1  0 |
| Poland  Institute of Mother and Child (Warsaw) | 6  6 | **4**  4 |
| Israel  Rambam Medical Center (Haifa)  Schneider Children's Medical Center (Petah Tikva) | 5  4  1 | **3**  3  0 |
| Spain  Hospital de Valdecilla, Santander  Virgen de las Nieves University Hospital (Granada)  Vall d'Hebron University Hospital (Barcelona)  Cruces University Hospital (Bilbao) | 4  1  1  1  1 | **3**  1  1  1  0 |
| The Netherlands  Princess Maxima Cancer Center (Utrecht) | 5  5 | **2**  2 |
| Czech Republic  Motol University Hospital (Prague)  Brno University Hospital | 3  2  1 | **3**  2  1 |
| United Kingdom  Great Ormond Street Hospital (London)  Birmingham Children's Hospital | 2  1  1 | **2**  1  1 |
| Austria  St Anna Children’s Hospital (Wien) | 2  2 | **2**  2 |
| Sweden  Karolinska University Hospital (Stockholm) | 2  2 | **2**  2 |
| Russia  Dmitriy Rogachev National Center (Moscow) | 2  2 | **2**  2 |
| Tunisia  Bab Saadoun University Hospital (Tunis) | 2  2 | **2**  2 |
| Algeria  Mustafa Hospital (Alger) | 1  1 | **1**  1 |
| Germany  Munster University Children’s Hospital | 1  1 | **0**  0 |
| **Total** | 74 | **60** |

**Table S2.** Differences between patients with and without response to MAPKi

|  | **Response**  **(n=37)** | **No response**  **(n=17)** | ***p*-value** |
| --- | --- | --- | --- |
| Median age at ND diagnosis (m) | 72 (41-98) | 95 (50-122) | 0.372 |
| Median time from LCH to ND diagnosis (m) | 41 (11-74) | 73 (22-84) | 0.273 |
| Median time from ND to MAPKi start (m) | 27 (10-64) | 19 (2-86) | 0.554 |
| Type of inhibitor  Vemurafenib (n=9)  Dabrafenib (n=22) | 7 (78)  15 (68) | 2 (22)  7 (32) | 0.655 |
| Median Florent score pre-treatment | 7 (2-12) | 6 (1-11) | 0.515 |
| Florent score pre-treatment  >4 (n=26)  <4 (n=27) | 18 (69)  20 (74) | 8 (31)  7 (26) | 0.835 |
| Median MRI score pre-treatment | 5 (3-7) | 6 (3-9) | 0.585 |
| MRI score pre-treatment  >6 (n=16)  <6 (n=38) | 11 (69)  27 (71) | 5 (31)  11 (29) | 0.963 |
| Median treatment duration (m) | 33 (14-66) | 26 (18-57) | 0.662 |

Data are n (%) and median (IQR)

*Abbreviations: LCH, Langerhans cell histiocytosis; ND, neurodegeneration; MAPKi, MAPK inhibitors*

**Figure S1.** Florent scale for clinical assessment


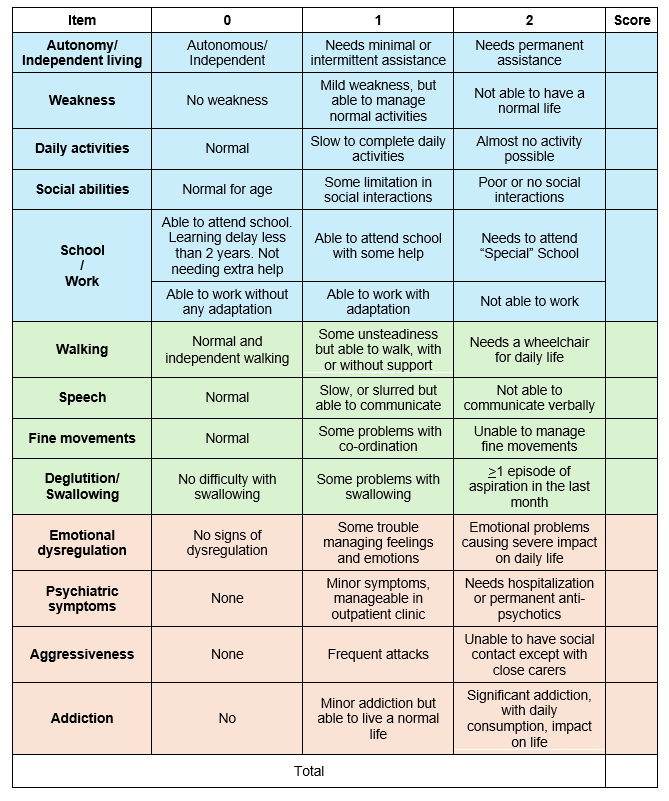


**Figure S2.** Modified Prosch scale for magnetic resonance imaging assessment


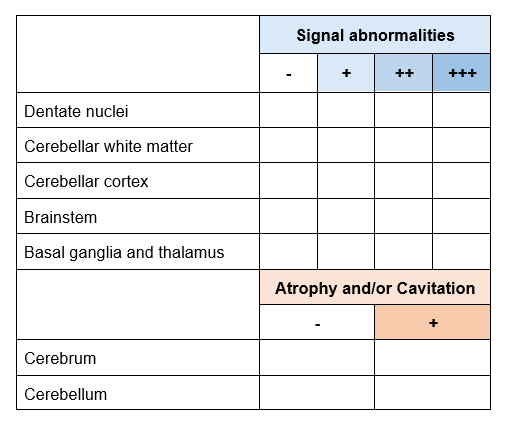


**Figure S3.** Survival probability


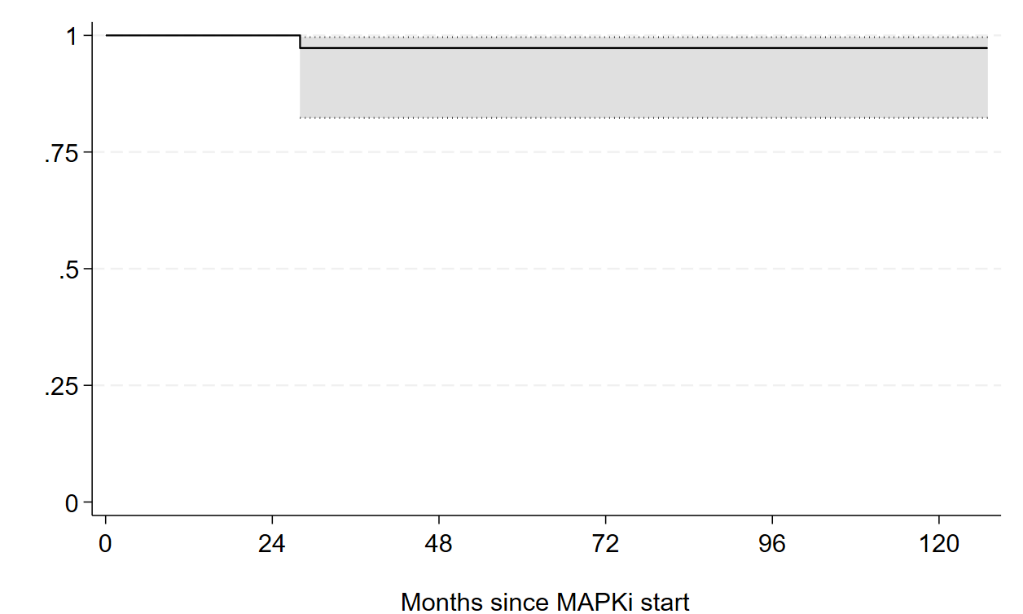

Supplement: Supplementary file 1 — Supporting Information. [file HEM3-10-e70392-s001.docx]
